# Editorial: Translation Regulation and Protein Folding

**DOI:** 10.3389/fpls.2022.858794

**Published:** 2022-03-02

**Authors:** M. Mar Castellano, Alejandro Ferrando, Markus Geisler, Hans-Peter Mock, Alfonso Muñoz

**Affiliations:** ^1^Centro de Biotecnología y Genómica de Plantas (UPM-INIA, CSIC), Campus de Montegacendo (UPM), Madrid, Spain; ^2^Instituto de Biología Molecular y Celular de Plantas CSIC-Universidad Politécnica de Valencia, Valencia, Spain; ^3^Department of Biology, University of Fribourg, Fribourg, Switzerland; ^4^Leibniz Institute of Plant Genetics and Crop Plant Research (IPK), Gatersleben, Germany; ^5^Departamento de Botánica, Ecología y Fisiología Vegetal, Universidad de Córdoba, Campus de Rabanales, Córdoba, Spain

**Keywords:** translation, protein folding, ribosomal protein (RP), translation regulation, cyclophilin, HSP70-HSP90 organizing protein, ER function, insertion into the thylakoid membrane

Proteins are the end products of the decoding process and constitute main structural elements and catalysts of the biochemical reactions in the cell. Proteins are synthetized during translation and in order to achieve their functional structure are folded. In this Research Topic, we aim at providing an update on the recent progress made in these two fundamental processes in plants.

Ribosomes constitute the translational machinery that, functioning as peptidyl transferases, decode mRNA information into polypeptides. The plant cytosolic ribosome is composed of 81 ribosomal proteins (RPs) that play essential roles in ribosome biogenesis and translation (Barakat et al., 2001; Chang et al., 2005; Wilson and Doudna Cate, 2012). Besides their essential role in protein synthesis, it is widely accepted that RPs also play ribosome-independent functions in different eukaryotes, including plants (Zhou et al., 2015; Xiong et al.). Xiong et al. review the role of RPs in non-related translational processes, such as transcriptional regulation, miRNA biogenesis, rDNA transcription, or protein and RNA folding. Remarkably, plants, compared to other eukaryotes, show a high complexity of the RP gene families, which usually encompass two to seven genes coding for multiple paralogs of each ribosomal protein (Barakat et al., 2001; Browning and Bailey-Serres, 2015). Since it is possible that different RP paralogs have undergone functional specialization, the study of whether these proteins have acquired novel extra-ribosomal functions is extraordinarily attractive in plants. In addition, the selective incorporation of these paralogs to the ribosome is also emerging as a potential mechanism of translation regulation which, along with others, may allow plants to adapt protein synthesis to their special growing characteristics (Castellano and Merchante, 2021). One of these characteristics is that plants are sessile organisms that must cope with the adverse environmental conditions in order to survive. Regarding this aspect, in this issue, Salazar-Diaz et al. describe that the Arabidopsis translation initiation factors eIF4E and eIFiso4E factors are involved in plant responses to cold and suggest that this role could be due to the selective translation of specific cold stress-related mRNAs. Despite the fact that more work should be done to fully elucidate the role of eIF4E isoforms in response to cold, this may constitute an additional example of the relevance of translational control and of selective translation in plant adaptation to the environment.

During translation, amino acids are coupled via peptide bonds to create the polypeptide chains, which are exposed to the solvent and begin their folding. This latter is an important process, since folding is determinant for protein structure and functionality. Most peptide bonds attain a *trans* conformation (Pauling et al., 1951; Ramachandran and Sasisekharan, 1968). However, some of the peptide bonds preceding prolines adopt the *cis* configuration, which usually introduces bends and decreases protein stability. In such a way, in many cases proper folding of these proteins requires a *cis* to *trans* isomerization of peptide bonds, an action carried out by peptidyl-prolyl *cis-trans* isomerases (PPIases) (Fischer and Aumüller, 2003). In this context, Singh et al. review the current knowledge about plant cyclophilins, proteins that bind cyclosporin A (CsA) and usually display PPIase activity, although some show an additional chaperone activity. This review reveals the extraordinary complexity, in terms of number of genes and domains, of the different cyclophilin families in plants. These characteristics, along with the localization of different cyclophilins in different compartments of the cell and their transcriptional regulation under different stress conditions, highly suggest that these proteins could be involved in multiple aspects of plant development and environmental responses (Singh et al.).

In addition to the involvement of the PPIases in protein folding, this topic also gathers information about different aspects of the quality control (QC) in plants. This system, through the action of multiple chaperones and co-chaperones, assists the folding of the nascent proteins (in those cases in which their structure is not achieved spontaneously) and assures that those proteins that have not achieved the correct conformation are degraded by the proteasome. In this context, the work by Toribio et al. compiles the recent advances in the knowledge of the plant family of co-chaperones HSP70-HSP90 organizing proteins (HOPs) and highlights the important role of these proteins in response to multiple stresses. Moreover, this work discusses the possibility that, HOPs, along with HSP90, play a relevant role, in different developmental programs, an aspect that is becoming to be elucidated (Di Donato and Geisler, 2019; Muñoz et al., 2021). In addition, in this topic, Reyes-Impellizzeri and Moreno review the involvement of the different components of the ER quality control (ERQC), the ER-associated degradation (ERAD) and the unfolded protein response (UPR) in abiotic stress (Reyes-Impellizzeri and Moreno). Interestingly, the two latter works agree on the need to identify the targets/clients of the chaperones and co-chaperones to fully understand the essential role of protein folding in these processes.

Plants are photosynthetic organisms that carry out the photosynthesis in the chloroplast. The proteome of the chloroplast encompasses ~3,000 proteins, most of which are nucleus-encoded and post-translationally imported into the organelle. Nevertheless, some crucial components of the photosynthesis machinery are encoded in the chloroplast genome and are translated by chloroplast ribosomes (Zoschke and Bock, 2018). In this issue, Ackermann et al. tackle the co-translational mechanism that allows the targeting of the chloroplast-translated proteins to the thylakoid membrane. These authors describe that the chloroplast ribosomal protein uL4c interacts with the thylakoid insertase Alb3 and suggest that this interaction could allow efficient processing of ribosome-nascent polypeptide chains during co-translational membrane insertion. Interestingly, this interaction seems to have evolved during the adaptation of plants from aquatic to land-based life.

Altogether, the articles included in this topic highlight the relevance of translation and protein folding in plant development and growth. Based on the rapid development of the field, we envision that new interesting findings will contribute to our understanding of these important, but quite unknown, regulatory processes.

## Author Contributions

AM and MMC wrote the final version of this editorial. All authors contributed to the article and approved the submitted version.

## Conflict of Interest

The authors declare that the research was conducted in the absence of any commercial or financial relationships that could be construed as a potential conflict of interest.

## Publisher's Note

All claims expressed in this article are solely those of the authors and do not necessarily represent those of their affiliated organizations, or those of the publisher, the editors and the reviewers. Any product that may be evaluated in this article, or claim that may be made by its manufacturer, is not guaranteed or endorsed by the publisher.
